# Why do lesions in the rodent anterior thalamic nuclei cause such severe spatial deficits?

**DOI:** 10.1016/j.neubiorev.2014.08.013

**Published:** 2015-07

**Authors:** John P. Aggleton, Andrew J.D. Nelson

**Affiliations:** School of Psychology, Cardiff University, Tower Building, 70 Park Place, Cardiff CF10 3AT, South Glamorganshire, Wales, UK

**Keywords:** Alternation, Amnesia, Direction, Fornix, Learning, Mammillary bodies, Memory, Navigation, Space, Thalamus

## Abstract

Lesions of the rodent anterior thalamic nuclei cause severe deficits to multiple spatial learning tasks. Possible explanations for these effects are examined, with particular reference to T-maze alternation. Anterior thalamic lesions not only impair allocentric place learning but also disrupt other spatial processes, including direction learning, path integration, and relative length discriminations, as well as aspects of nonspatial learning, e.g., temporal discriminations. Working memory tasks, such as T-maze alternation, appear particularly sensitive as they combine an array of these spatial and nonspatial demands. This sensitivity partly reflects the different functions supported by individual anterior thalamic nuclei, though it is argued that anterior thalamic lesion effects also arise from covert pathology in sites distal to the thalamus, most critically in the retrosplenial cortex and hippocampus. This two-level account, involving both local and distal lesion effects, explains the range and severity of the spatial deficits following anterior thalamic lesions. These findings highlight how the anterior thalamic nuclei form a key component in a series of interdependent systems that support multiple spatial functions.

## Highlights

• A dual hypothesis is introduced to explain the importance of these thalamic nuclei.
• ATN are vital for multiple spatial functions.
• ATN damage disrupts processing across distal limbic sites.
• Distal pathology caused by ATN damage disrupts plasticity and metabolic activity.
• ATN lesion effects reflect both their intrinsic importance and distal dysfunctions.

## 1 Introduction

On reflection, it is remarkable that lesions in the anterior thalamic nuclei (ATN) produce severe, long lasting deficits in rodents on a such wide range of spatial memory tests (e.g., Aggleton et al., 1995a, 1996; Aggleton and Sahgal, 1993; Beracochea and Jaffard, 1994; Byatt and Dalrymple-Alford, 1996; Célérier et al., 2000; Dumont et al., 2014a; Loukavenko et al., 2007; Mair et al., 2003; Mitchell and Dalrymple-Alford, 2005, 2006; Sutherland and Rodriguez, 1989; Sziklas and Petrides, 1999; Van Groen et al., 2002a). There are many different forms of spatial learning, which are supported by a multiplicity of brain sites (Mizumori et al., 2000; Taube, 2007; Moser et al., 2008), so why should these thalamic nuclei be so important? The finding is all the more extraordinary as animals can often switch between different strategies, thereby, counteracting impairments to specific spatial abilities. The implication is, therefore, that the ATN must either be critical for a range of spatial processes or that these thalamic lesions disrupt a fundamental process upon which multiple forms of spatial learning then depend. An example of the second account might be that the ATN are required for integrating intrinsic body signals with extrinsic spatial information.

This review provides a two-level explanation for why anterior thalamic lesions have such disruptive effects on spatial learning. The first level concerns the loss of functions provided by the anterior thalamic nuclei themselves, with the conclusion that these nuclei have multiple functions that contribute to effective spatial learning. The second level concerns the loss of function following ‘covert pathologies’ found in sites distal to the anterior thalamic nuclei, sites that normally support spatial learning. Because these same distal sites also appear to support multiple aspects of spatial learning, the impact of ATN lesions across a range of spatial processes is further exacerbated.

Reflecting this two-level account, the early sections of this review are concerned with the impact of lesions in the anterior thalamic nuclei. Some additional evidence comes from studies into the effects of mammillary body lesions as these hypothalamic nuclei have very dense projections focussed on the anterior thalamic nuclei. Particular attention is given to tests of T-maze alternation. This spatial test is readily learnt, has been used in many experiments, and is highly sensitive to anterior thalamic damage. Although spatial alternation is a test of ‘working memory’ (see Section 2.1), this feature is in itself not critical as anterior thalamic nuclei lesions also impair ‘reference memory’ tasks, such as learning the location of a submerged platform in the Morris water pool (Sutherland and Rodriguez, 1989; Warburton and Aggleton, 1999; Warburton et al., 1999; Wolff et al., 2008a,b). Later sections are concerned with the visualisation and mapping of neuronal dysfunctions beyond the thalamus, caused by anterior thalamic lesions. The implication from these latter experiments is that the spatial deficits following anterior thalamic lesions reflect a much broader array of brain dysfunctions than those evident from classic histological methods.

## 2 T-maze alternation and the anterior thalamic nuclei (ATN)

### 2.1 The reinforced T-maze alternation task

**Fig. 1:**
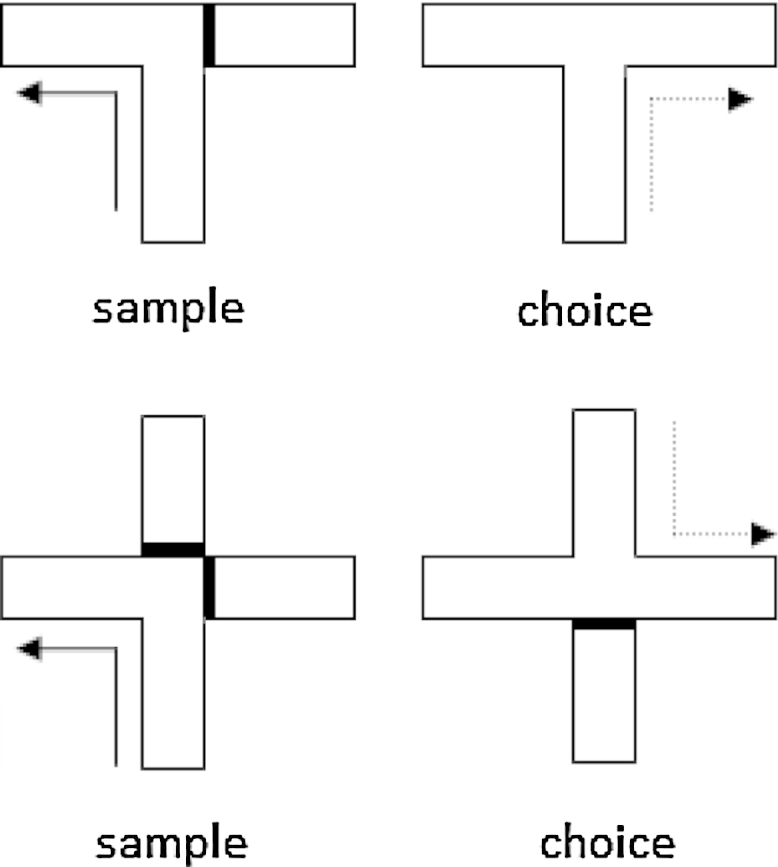
T-maze (upper) and cross-maze (lower) arrangements used to test spatial alternation. The bold line depicts the barrier used on sample runs (solid arrow) to control the arm choice by the animal. The dashed arrow shows the correct arm in the choice test. The cross-mazes show how opposing start positions can be used for the sample run and choice test in order to disrupt egocentric and directional alternation.

One of the most widely studied spatial abilities in rodents is T-maze alternation (Dember and Fowler, 1958; Dudchenko, 2001; Lalonde, 2002). Each alternation trial is in two stages (Fig. 1). For reinforced alternation, used in the large majority of lesion studies, the rat or mouse first runs up the stem of the T-maze and is only allowed to enter one of the two cross arms (‘sample’ run). In that arm the animal receives a reward. Next, the rodent is picked up and placed back at the start of the T-maze and, after a delay, is allowed a free choice between the two cross arms (‘choice’ test). The rodent is rewarded for selecting the arm opposite to that entered in the sample run, i.e., nonmatching-to-place. The choice of sample arm (and, hence, the rewarded arm on the choice test) is independent of the preceding trial. For this reason the task taxes ‘working memory’ (Olton et al., 1979).

It has been repeatedly shown that T-maze alternation is highly sensitive to ATN damage in rats (Aggleton et al., 1995a, 1996, 2009; Dumont and Aggleton, 2013; Loukavenko et al., 2007; Warburton and Aggleton, 1999; Warburton et al., 1997, 1999) and mice (Beracochea and Jaffard, 1994; Célérier et al., 2000). Following ATN lesions in rats, alternation performance often starts close to chance levels. Although an improvement is sometimes seen, the animals fail to reach normal levels of accuracy. Even when rats with ATN lesions are given environmental enrichment, which improves spatial alternation performance, the rats remain impaired with respect to their enriched controls (Loukavenko et al., 2007). Likewise, rats trained on T-maze alternation prior to their ATN lesions are still severely impaired when subsequently re-tested on spatial alternation after surgery (Warburton et al., 1999). This robust alternation impairment is all the more striking given that the task is so easy for intact rats to solve, sometimes resulting in near-ceiling levels of performance. An analysis of this task should, therefore, cast light on the wider spatial functions of the anterior thalamic nuclei.

It has long been known that rats will spontaneously avoid the arm of a T-maze last visited, instead preferring the novel arm or the arm that had been visited longer ago in time (Dember and Fowler, 1958; Dudchenko, 2001). This spontaneous preference reflects a bias to approach stimuli that are novel (Dember, 1956; Kivy et al., 1956). It is clear, however, that anterior thalamic lesions do not affect the tendency to detect and approach novel stimuli per se, as shown when testing object recognition memory (Mitchell and Dalrymple-Alford, 2005; Warburton and Aggleton, 1999; Wilton et al., 2001b). A different issue relates to the likelihood that testing in the T-maze, at least initially, may be anxiogenic. If anterior thalamic lesions affect anxiety then it is possible that such nonspatial changes could indirectly disrupt spatial memory. In fact, when tested in an elevated plus-maze, rats with anterior thalamic lesions appear to show reduced anxiety, as measured both by behaviour and by levels of corticosterone (Dupire et al., 2013). The implication is, therefore, that the lesion-induced alternation deficits principally arise from the spatial demands of the task.

Rodents potentially solve spatial alternation tasks using a number of different strategies. These include: (1) place alternation, i.e., avoiding returning to a place as determined by its allocentric, i.e., extra-maze, cues, (2) intra-maze cue alternation, i.e., avoiding an arm because it contains local cues, such as olfactory signals, associated with the most recent arm visit, (3) direction alternation, i.e., using a directional bearing in the test room around which to alternate, akin to alternating around a fixed compass heading, and (4) egocentric alternation, i.e., using the direction of body turns to make opposing movements, so turning left around the body axis then turning right (or vice versa). While direction alternation and egocentric alternation may sound similar, they can be distinguished by changing the orientation of the T-maze between the sample and choice trials (Dudchenko, 2001). This manipulation should disrupt direction alternation but not egocentric alternation. Rather, it is place alternation and direction alternation that may seem most alike given their shared use of distal spatial cues. Even so, place alternation is assumed to depend on identifying unique locations while direction alternation uses heading cues that will remain the same in spite of a change in immediate location. Many decades of behavioural testing have shown that rats are able to use strategies 1–3 (Dudchenko, 2001), but often seem unable to use egocentric information for working memory problems, unless the retention intervals are exceptionally short (Baird et al., 2004; Futter and Aggleton, 2006; Pothuizen et al., 2008; but see Mitchell and Dalrymple-Alford, 2006). The situation for reference memory tasks in cross or T-mazes is very different, where response based learning involving egocentric information can dominate, especially after extended training (e.g. Packard and McGaugh, 1996). Such egocentric tasks are not, however, affected by anterior thalamic lesions (Warburton et al., 1999).

The availability of multiple alternation strategies implies that lesion effects should often be relatively minor as the animal can employ any strategies spared by the surgery. For this reason, it is striking that T-maze alternation levels after ATN lesions in rats often start close to chance, with group means typically between 50 and 65%, (Aggleton et al., 1995a, 1996, 2009; Loukavenko et al., 2007; Warburton and Aggleton, 1999; Warburton et al., 1997, 1999), often remaining little changed throughout testing (Aggleton et al., 2009; Loukavenko et al., 2007; Warburton and Aggleton, 1999; Warburton et al., 1997, 1999). In some studies of ATN lesions there is clearer evidence of improvement with training, although the rats still remain impaired (Loukavenko et al., 2007, enriched housed group). In mice, the alternation deficit after ATN lesions appears less severe (Beracochea and Jaffard, 1994; Célérier et al., 2000). Even so, the overall scale of these deficits, especially in rats, is striking when it is remembered that the test animal is constrained by the apparatus so that it can only turn right or left. This arrangement contrasts with the added navigational demands posed by tasks such as the Morris water maze. Furthermore, severe alternation deficits are found even though the choice arms are set at 180° to each other, i.e., any spatial discrimination is made as simple as possible.

There are obvious similarities between reinforced T-maze alternation and the standard radial-arm maze task (Olton et al., 1979). Both are tests of spatial working memory that involve nonmatching-to-place. While the choice locations in the radial-arm maze should be harder to distinguish than those in a T-maze, as there are more of them, there is arguably less proactive interference in the radial-arm maze for any given location as rats typically receive just one completed test per session. Unsurprisingly, working memory performance in the radial-arm maze is disrupted by anterior thalamic damage (Alexinsky, 2001; Byatt and Dalrymple-Alford, 1996; Mitchell and Dalrymple-Alford, 2005; Sziklas and Petrides, 1999; Warburton et al., 2001; but see Beracochea et al., 1989; M’Harzi et al., 1991). Findings from radial-arm maze studies are considered in this review when they provide additional insights.

### 2.2 The pattern of T-maze alternation deficits after anterior thalamic damage

One way to solve a spatial alternation task is to nonmatch-to-place. The question is, therefore, whether ATN lesions bring about a failure to distinguish the choice locations. Given that alternation deficits can occur on the first trial of a session, when temporal separation would be easiest (see below), it would seem most likely that place learning is substantially disrupted by ATN lesions. More direct evidence for a place learning deficit comes from the consistent ATN lesion deficits found in water-maze location tasks (Sutherland and Rodriguez, 1989; Warburton and Aggleton, 1999; Warburton et al., 1997, 1999; Wolff et al., 2008a,b; Moreau et al., 2013). These water-maze deficits are characterised by an increased latency to find the escape platform and inaccurate searching on probe trials when the escape platform is removed. While the standard fixed location protocol in the water maze has aspects of a matching-to-place task, it remains a reference memory problem, unlike T-maze alternation.

There are, however, several reasons to suppose that a deficit in allocentric place learning is unlikely to provide a complete explanation for the alternation deficit. Rats with ATN lesions that were impaired in acquiring a standard water-maze task, could still distinguish the correct quadrant in the water-maze in a final probe test, performing at a similar level to the controls (Warburton and Aggleton, 1999). Despite this place learning, the same rats performed at around 65% on a subsequent T-maze test (Warburton and Aggleton, 1999), where only the two halves of a room need to be distinguished. In addition, rats with ATN lesions were only mildly impaired at acquiring a go/no go discrimination between two different test locations when each location was approached from a constant, but different direction (Dumont et al., 2014a), akin to what happens in the T-maze. Likewise, rats with ATN lesions could recognise the correct corner in a square pool, defined by the different patterns on the walls in that corner (Dumont et al., 2014b), though the same rats were severely impaired on T-maze alternation (Dumont and Aggleton, 2013). Finally, rats with ATN lesions are also impaired on a delayed-nonmatching to lever task (Aggleton et al., 1991), a spatial task that can be dissociated from T-maze alternation (Aggleton et al., 1995a) showing that these nuclei are involved with multiple classes of location information. Thus, although ATN lesions impair place learning, they appear to have additional effects that impact on T-maze alternation.

To gain a better insight, some researchers have used a cross-maze for alternation studies (Fig. 1). This apparatus makes it possible to look at the consequences of switching the start arm by 180°, e.g., approach from the South on the sample run but from the North on the choice test, so presumably disrupting direction alternation and effectively stopping an egocentric solution (Fig. 1). This switch has proved to be particularly challenging for rats with ATN lesions as their performance on the ‘opposite’ start condition is at chance (Loukavenko et al., 2007; Warburton et al., 1997). Performance on the ‘same’ start condition was superior, although the rats with ATN lesions remained impaired on this easier condition (Loukavenko et al., 2007; Warburton et al., 1997). A similar pattern has been seen after mammillary body lesions (Neave et al., 1997).

This pattern of ATN lesion deficits in the cross-maze is intriguing as nonmatching-to-place or nonmatching-to-intra-maze cues should remain equally accurate with either ‘same’ or ‘opposite’ arm starts, i.e., they should not be disrupted by the switch in start position. Thus, the switch effects should logically reflect the disruption of directional or egocentric alternation. In fact, as already noted, rats normally fail to use egocentric information for spatial nonmatching (Baird et al., 2004; Futter and Aggleton, 2006; Pothuizen et al., 2008; Mitchell and Dalrymple-Alford, 2006). It remains, however, possible that ATN lesions force an unusual reliance on egocentric information (Loukavenko et al., 2007) given that rats with ATN lesions seem unimpaired at learning reference memory egocentric discriminations (Warburton et al., 1997; Wolff et al., 2008a; see also Mitchell and Dalrymple-Alford, 2006). A second possibility is that rats with ATN lesions use spared direction information in a standard T-maze, so explaining the disruption caused by ‘opposite’ starts in a cross-maze. This suggestion is also problematic given the loss of head direction cells by ATN lesions (see Section 2.3), potentially reflected by the deficits seen on tests of path integration (Frohardt et al., 2006), as well as evidence that both mammillothalamic tract lesions and retrosplenial cortex lesions disrupt direction-based alternation in a cross-maze (Pothuizen et al., 2008; Vann, 2013).

An alternative explanation is, therefore, required for the increased deficit found when switching the start position in a cross-maze deficit. One possibility is that moving to the opposite start position is unduly disorienting for rats with ATN lesions as they do not identify the new location as part of the same apparatus and consequently treat the choice test as if it were a new trial, i.e., a sample run. In contrast, control rats learn the overall layout of the cross-maze and so appreciate the continuity of the test conditions (Still and Macmillan, 1969).

An integral feature of T-maze alternation is the increasing level of proactive interference as a test session progresses. This occurs because of growing competition between information from the last sample run and the information from preceding trials. Consequently, performance by control animals may be best on the very first trial of a session (Beracochea and Jaffard, 1994; Dumont et al., 2014b), when interference effects are least. Interference can be deliberately increased by using a continuous alternation design in which there is no separate sample run and the inter-trial intervals are kept short (Aggleton et al., 1995a). Although this manipulation increases task difficulty, there is no evidence that it is excessively sensitive to anterior thalamic damage (Aggleton et al., 1995a). Furthermore, when rats are given spaced trials, e.g., inter-trial intervals of 4 min, ATN lesions remain highly disruptive, even though proactive interference is presumably reduced (e.g., Aggleton et al., 1995a, 1996, 2009; Warburton et al., 1997, 1999). Indeed, ATN lesion deficits are still found on the very first trial of each session (e.g., Dumont et al., 2014b), when interference should be close to a minimum.

The conclusion is, therefore, that heightened sensitivity to interference is not sufficient to explain the full impact of anterior thalamic lesions. Even so, there are reasons to believe that sensitivity to proactive interference contributes to the alternation deficit. These reasons include findings from nonspatial studies of recency memory, which show that ATN lesions can sometimes disrupt the ability to distinguish between items on the basis of their serial order (Dumont and Aggleton, 2013; Wolff et al., 2006; but see Mitchell and Dalrymple-Alford, 2005). Those studies that have examined recency judgements when the rat is removed from the apparatus between the presentation of the two objects to be subsequently discriminated have reported no deficit (Mitchell and Dalrymple-Alford, 2005; Dumont and Aggleton, 2013). In contrast, when the recency task involves objects (Dumont and Aggleton, 2013) or odours (Wolff et al., 2006) that are presented in a continuous series, so increasing interference, impairment are found. Finally, there is also evidence that mammillary body lesions in mice can spare alternation performance on trial one but impair later alternation trials in the same session (Beracochea and Jaffard, 1987). These findings all suggest that temporal confusions can exacerbate the spatial alternation deficits after ATN damage.

**Fig. 2:**
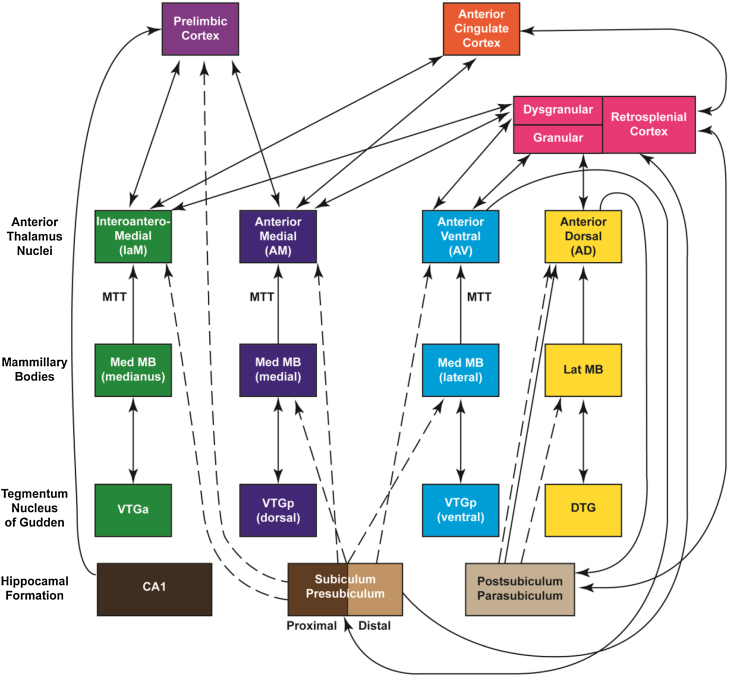
Schematic diagram illustrating how the rat hippocampal formation is associated with distinct sets of parallel, anterior thalamic connections. Connections conveyed via the fornix from the subiculum are shown with dashed lines. (Note that AD also receives nonfornical inputs from the postubiculum.) Double-headed arrows depict reciprocal connections. Abbreviations: DTG, dorsal tegmental nucleus of Gudden; MTT, mammillothalamic tract; VTGa, ventral tegmental nucleus of Gudden, pars anterior; VTGp, ventral tegmental nucleus of Gudden, pars posterior.

Intra-maze cues, including odour trails, may provide a basis for alternation behaviour (Douglas, 1966). As such cues are non-spatial it might be assumed that they could still be effectively used for alternation by rats with ATN lesions. Indeed, there is evidence that rats with mammillothalamic tract lesions and with retrosplenial cortex lesions are unduly reliant on intra-maze cues for both T-maze alternation and radial-arm maze performance (Vann, 2013; Vann et al., 2003). The ability of rats with ATN lesions to use local context cues to guide other discriminations (Dumont et al., 2014a) would again make this plausible. If such cues are used, they can only provide a poor strategy given the low levels of performance seen by rats with ATN lesions, perhaps exacerbated by interference (temporal discrimination) deficits. While intra-maze cue usage could be tested by rotating the maze between sample and test, or by using pairs of T mazes, the fact that rats with ATN lesions start at such low levels of performance (Fig. 2) makes the interpretation of such manipulations difficult.

In T-maze alternation, there is always a retention interval between sample and test trials. As a consequence, alternation deficits could arise from faster forgetting of the relevant spatial information. In fact, studies with rats report severe ATN lesion deficits starting from the shortest delay tested, e.g., 10 s (Aggleton et al., 1995a, 1996, 2009; Loukavenko et al., 2007; Warburton et al., 1999, 2001) so that, although retention delays of up to 40 s have been examined (Warburton et al., 1997), any disruptive effect is difficult to interpret as the lesion and sham groups are not matched before the retention intervals are extended. Lesion studies in mice do, however, provide clearer evidence of delay-dependent alternation deficits following ATN damage (Beracochea and Jaffard, 1994; Célérier et al., 2000). In one study, alternation performance by mice with ATN lesions was matched to that of the controls at the shortest delay (30 s), but clear impairments were found with a 60 s delay (Célérier et al., 2000). In another study, a double sample run was used followed by delays of 5 min and then 6 h. The ATN lesions only impaired alternation performance after 6 h retention intervals (Beracochea and Jaffard, 1994). Finally, faster rates of forgetting after ATN lesions were seen for a nonmatching-to-sample problem that used levers in an operant box (Aggleton et al., 1991), where performance was unimpaired at the shortest delay but impaired as the retention interval increased.

When the relevant findings are amassed, it becomes difficult to identify a single task demand that is sufficient to account for the full alternation deficit after ATN lesions. Instead, evidence can be found to support an array of contributory deficits (place discrimination, temporal discrimination, direction learning, enhanced forgetting rates, and sensitivity to proactive interference). For this reason, the next two sections consider this apparent multiplicity of spatial deficits associated with ATN damage based on two different explanations.

The first explanation is that the three major ATN nuclei are responsible for different aspects of spatial processing and, thus, when all three are removed, the deficits become particularly wide ranging. The second explanation is that surgeries targeted at the anterior thalamic nuclei often unintentionally damage immediately adjacent thalamic nuclei, which then contribute to the spatial deficit. These two explanations are not mutually exclusive.

### 2.3 Are the different anterior thalamic nuclei (anteromedial, anterodorsal, anteroventral) responsible for different aspects of spatial processing?

Evidence that the three major anterior thalamic nuclei have different functions has been recently reviewed (Aggleton et al., 2010). In brief, initial support comes from their connectivity (Fig. 2), as the large majority of afferents to the three major nuclei arise from different cells, often with topographies that further distinguish their inputs (Wright et al., 2013). This anatomical organisation maintains the potential for different types of spatial information to be processed by different parts of the anterior thalamic nuclei (Aggleton et al., 2010). Arguably, the strongest evidence comes from comparisons of the electrophysiological properties of the anterior thalamic nuclei (Albo et al., 2003; Jankowski et al., 2013; Tsanov et al., 2011a; Vertes et al., 2001, 2004).

The anterodorsal thalamic nucleus and, to a lesser extent, the anteroventral thalamic nucleus contain head direction cells (Taube, 1995, 2007; Tsanov et al., 2011a). These cells provide compass-like information as they indicate heading direction independent of absolute location (Taube, 2007). Head direction information is likely to contribute to effective alternation behaviour (Pothuizen et al., 2008; Wilton et al., 2001b), though the loss of this information from the anterodorsal nucleus is not sufficient to explain the severe deficits seen after complete ATN lesions. This conclusion can be reached because of the relatively mild disruptive effects of lesions that target the anterodorsal nucleus, i.e., spare the anteromedial nucleus (Aggleton et al., 1986, see also Byatt and Dalrymple-Alford, 1996; Van Groen et al., 2002a). Likewise, lesions of the lateral mammillary nucleus produce only mild, transient alternation deficits (Vann, 2005, 2011), yet it is this nucleus that provides head direction information for the anterior thalamic nuclei. These relatively mild impairments are all the more striking because anterior thalamic lesions result in an absence of head direction activity in the hippocampal formation (Goodridge and Taube, 1997). At the same time, the presence of head direction cells in additional sites, including the retrosplenial cortex and nucleus reuniens (Cho and Sharp, 2001; Jankowski et al., 2014) may permit this information to assist alternation behaviour, despite anterior dorsal thalamic damage.

Recording studies indicate that 75% of cells in the anteroventral nucleus fire rhythmically with theta (Albo et al., 2003; Vertes et al., 2001, 2004). This finding has been interpreted as suggesting that a key property of this nucleus is the conveyance of theta to the hippocampal formation, with its potential involvement in optimising synaptic plasticity (Rutishauser et al., 2010). For this reason, the anteroventral nucleus has been described as providing a ‘return loop’ system, primarily involved in hippocampal processing (Aggleton et al., 2010). In contrast, only a small percentage of cells (6%) in the anteromedial nucleus show rhythmic theta firing (Albo et al., 2003). It has been suggested that the anteromedial nucleus forms part of a ‘feed-forward’ system that is primarily concerned with conveying integrated hippocampal–diencephalic signals to prefrontal areas to aid cognitive flexibility, executive function, and recency judgements (Aggleton et al., 2010). The implication is that the three major anterior thalamic nuclei have properties that reflect different, but complementary, contributions to spatial learning. In the case of alternation learning, a simplistic division would emphasise the anterodorsal nucleus for direction information, the anteroventral nucleus for place information, and the anteromedial nucleus for response flexibility and temporal information.

### 2.4 Are the effects of anterior thalamic nuclei lesions exacerbated by damage to adjacent sites?

**Fig. 3:**
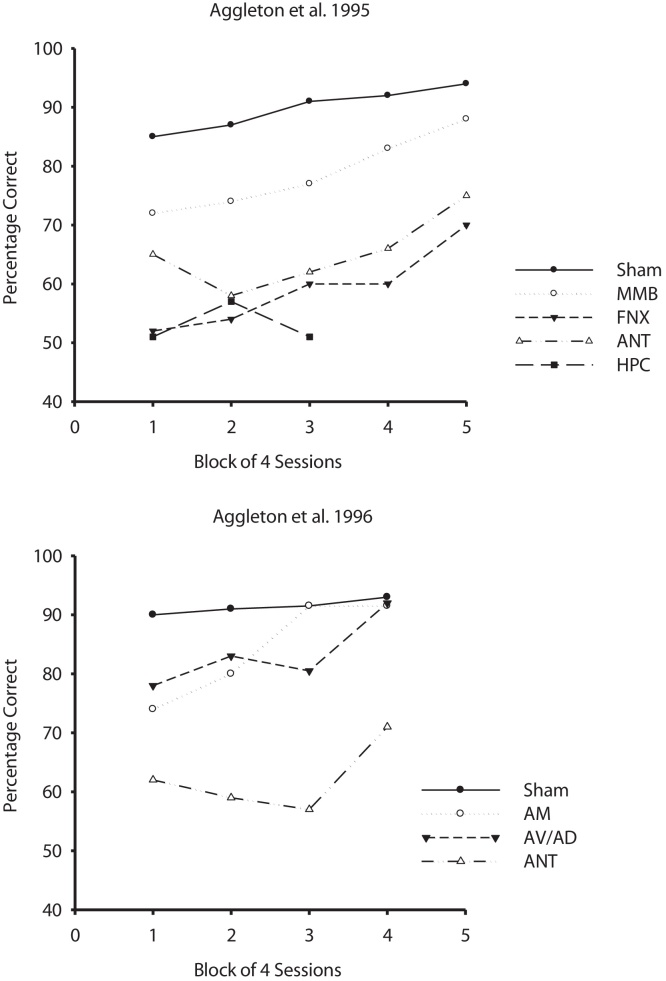
Performance of rats on reinforced T-maze alternation with lesions of the anterior thalamic nuclei (ANT) and closely related structures. The upper graph shows comparison performance (chance is 50%) from rats with fornix (FNX), mammillary body (MMB) and Sham lesions (Aggleton et al., 1995a,b), as well as rats with hippocampal (HPC) lesions (Aggleton et al., 1986). The lower graph compares the performance of rats with selective lesions centred in the anteromedial nucleus (AM), or the anteroventral and anterodorsal nuclei (AV/AD), as well as rats with combined lesions (ANT) involving all anterior thalamic nuclei (Aggleton et al., 1996). In all studies there was a retention interval of 10–15 s between the sample and choice run of each trial. The inter-trial interval was ∼4 min.

The second explanation for the multiplicity of lesion effects is that unintended damage beyond the anterior thalamic nuclei has often contributed to the pattern of spatial deficits. Those studies that have sought to confine lesions within either the anteromedial or the anterodorsal/anteroventral thalamic nuclei provide highly relevant evidence as the lesions are unusually small and, therefore, the least likely to impact on other nuclei (Fig. 3). These highly selective lesions show that the loss of the anterior thalamic nuclei is sufficient to impair spatial tasks, including T-maze alternation (Aggleton et al., 1986), as well as the radial-arm maze and Morris swim maze (Byatt and Dalrymple-Alford, 1996; Van Groen et al., 2002a). One study providing an exception involved small lesions centred in either the anteroventral nucleus or the anteromedial nucleus that appeared to spare continuous alternation in a T-maze (Greene and Naranjo, 1986). The sparing in this case may reflect the procedure, in which the rats ran back to the start point unaided, i.e., the rats were not carried, so potentially encouraging a figure of eight response. Other relevant data come from those studies in which the surgery targeted all of the anterior thalamic nuclei but the lesions appear to be unusually well confined within the target region. These studies still describe clear performance deficits on T-maze alternation (Aggleton et al., 1995a, 2009; Warburton et al., 1999—smaller lesion group).

At the same time, it is important to re-examine those studies with more extensive lesions of the rostral thalamus. Lesions of the anterior thalamic nuclei may unintentionally involve a number of adjacent thalamic nuclei, including the laterodorsal nucleus, medial dorsal nucleus, rhomboid nucleus, nucleus reuniens, parts of the rostral reticular nucleus, and the parataenial nucleus. Some studies have specifically observed that such unintended cell loss can increased the scale of the anterior thalamic lesion deficit (Warburton et al., 1997, 1999). Of the vulnerable nuclei, the laterodorsal nucleus stands out as it shares many of the anatomical properties of the anterodorsal thalamic nucleus (Van Groen and Wyss, 1992b) and, like the anterodorsal nucleus, contains head direction cells (Taube, 2007). Combined lesions involving both the anterodorsal nucleus and the laterodorsal thalamic nucleus produce marked alternation deficits (Wilton et al., 2001b), suggesting that encroachment into the laterodorsal nucleus can, indeed, exacerbate spatial deficits (Warburton et al., 1997; see also Van Groen et al., 2002b).

The impact of lesions in the medial dorsal thalamic nucleus on T-maze alternation has been examined in several studies, and while damage to this nucleus mimics some of the effects of prefrontal cortex lesions (Cross et al., 2013; Hunt and Aggleton, 1998), it does not appear to produce selective spatial deficits (Hunt and Aggleton, 1991, 1998). Furthermore, a comparison of ATN lesions that did or did not extend into the rostral medial dorsal nucleus failed to find a difference in the severity of the alternation deficit (Warburton and Aggleton, 1999). A different result was found, however, when the comparison involved ATN lesions that additionally extended into midline thalamic nuclei (including reuniens and rhomboid) and the rostral intralaminar nuclei. Now, the extra damage was associated with more severe T-maze alternation deficits (Warburton et al., 1999). Although these larger lesions were also more likely to produce more complete ATN lesions, there is growing evidence for a contribution from these additional thalamic nuclei, including a role in strategy learning (Cassel et al., 2013; Cain et al., 2006).

Nucleus reuniens is of particular interest because of its dense, direct connections with both the prefrontal cortex and hippocampus (Vertes et al., 2006; Prasad and Chudasama, 2013). Furthermore, this nucleus contains head direction cells (Jankowsky et al., 2014). Lesion evidence indicates that nucleus reuniens (possibly along with the adjacent rhomboid nucleus) can influence spatial learning in the radial-arm maze (Davoodi et al., 2009; Hembrook and Mair, 2011) and water-maze (Dolleman-van der Weel et al., 2009; Loureiro et al., 2012). Joint inactivation of reuniens and rhomboid also impairs a working memory version of a spatial conditional task run in a T-maze (Hallock et al., 2013). These findings all suggest that damage to nucleus reuniens (and possibly the rhomboid nucleus) could add to the spatial deficits associated with ATN damage (Cassel et al., 2013).

Of the remaining candidate sites, only a few lesion studies have examined the impact of lesions in the rostral reticular nucleus lesions on spatial learning. In one of these studies (M’Harzi et al., 1991) reticular nucleus lesions were associated with working memory deficits in the radial-arm maze, while surprisingly anterior thalamic lesions had little impact. In a second study (Wilton et al., 2001a), rostral reticular nucleus lesions led to a transient T-maze alternation deficit that rapidly disappeared. The same rats then seemed unaffected in the radial-arm maze or Morris water maze (Wilton et al., 2001a). The parataenial nucleus remains under-explored, principally because of the difficulty in making selective manipulations in this small nucleus.

Two final sites should also be briefly considered. When making stereotaxic rostral thalamic lesions, tracts typically go through the fornix to reach the thalamus. For this reason, the impact of injecting NMDA into the fornix itself was assessed (Warburton et al., 1997) but spatial alternation deficits were not observed. Likewise, when lesions have been made in the thalamic intralaminar nuclei, which are caudal to the anterior thalamic nuclei, spatial learning seems largely unaffected (Moreau et al., 2013; Wolff et al., 2008a).

The conclusion is that additional thalamic damage may well add both quantitatively and qualitatively to the spatial learning deficits following lesions targeted at the anterior thalamic nuclei. At the same time, there is convincing data to believe that anterior thalamic nuclei damage is sufficient to markedly disrupt spatial alternations.

## 3 The anterior thalamic nuclei as part of a distributed network

The message that ATN damage can disrupt multiple aspects of spatial processing implies a variety of interactions with other sites making quite different contributions to spatial learning and memory. This possibility can be explored by examining the impact of lesions in sites interconnected with the anterior thalamic nuclei.

### 3.1 Interlinked systems for spatial alternation revealed by lesion studies

Performance on T-maze alternation is highly sensitive to hippocampal lesions (e.g. Aggleton et al., 1986; Bannerman et al., 1999; Dudchenko et al., 2000). Accuracy may remain at chance levels after surgery (Fig. 3). Alternation deficits are also seen after fornix lesions (e.g., Aggleton et al., 1995a, 2009; Bussey et al., 2000; Neave et al., 1994, 1997; Warburton et al., 1998). In those studies that have looked at the effects of both ATN and fornix lesions (Fig. 3), the severity of the alternation deficits is often comparable (Aggleton et al., 2009; Warburton et al., 1997, 1999). These findings are intriguing given the dense hippocampal projections to the anterior thalamic nuclei, many of which rely on the fornix (Swanson and Cowan, 1977). As the fornix contains many other connections, it is necessary to use disconnection methods to test the idea that the hippocampal and anterior thalamic spatial impairments are functionally linked.

Crossed unilateral lesions involving the hippocampus and anterior thalamic nuclei produce clear deficits on T-maze alternation, strongly supporting an inter-dependent function (Warburton et al., 2001). These findings are supported by the outcome of surgeries involving crossed unilateral lesions of the anterior thalamic nuclei and fornix (Warburton et al., 2000). Interestingly, such combined surgeries only become markedly disruptive to T-maze alternation when the lesions are placed to maximise the disconnection of thalamic inputs (Warburton et al., 2000). A further feature of both ATN-hippocampus disconnection studies is that the lesioned rats showed clear improvements over spatial alternation training (Warburton et al., 2000, 2001), which may reflect the increased recruitment of spared, crossed pathways.

A part of this disconnection effect is presumably the loss of hippocampal (postsubicular) head direction information after anterior thalamic lesions (Goodridge and Taube, 1997). Related experiments have also shown that anterior thalamic lesions, but not mammillary body lesions, degrade the spatial coherence and information content from hippocampal place fields (Calton et al., 2003; Sharp and Koester, 2008). Perhaps more surprising is evidence that the anterior thalamic nuclei can affect hippocampal neurogenesis in the rodent brain. It has been reported that high frequency stimulation in the anterior thalamic nuclei can increase neurogenesis in the dentate gyrus (Encinas et al., 2011; Toda et al., 2008) and aid the performance of memory tasks (Hamani et al., 2011). Furthermore, pharmacological lesions of the anterior thalamic nuclei suppress hippocampal neurogenesis (Kuramoto et al., 2009). These findings are particularly intriguing given the considerable evidence that hippocampal neurogenesis has an important role in learning and memory (Deng et al., 2010). Such findings relate to the impact of ATN lesions on distal sites, including the hippocampus (see Section 4).

**Fig. 4:**
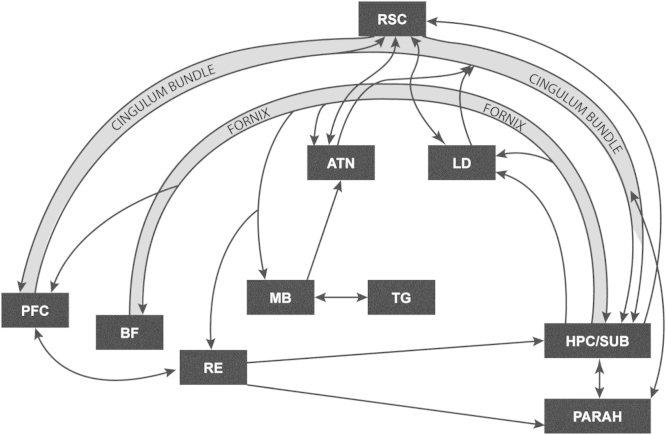
Schematic diagram showing some of the interconnections between sites implicated in spatial learning in rodents and anterograde amnesia in humans. Abbreviations: ATN—anterior thalamic nuclei; BF—basal forebrain (including septum and diagonal band); HPC/SUB—hippocampal formation (including subiculum); LD—laterodorsal thalamic nucleus; MB—mammillary bodies; PARAH—parahippocampal region; PFC—prefrontal cortex; RE—nucleus reuniens of the thalamus; RSC—retrosplenial cortex; TG—tegmental nucleus of Gudden.

In view of their dense connections with both the anterior thalamic nuclei and the hippocampus (Fig. 4), it is not surprising that lesions of the mammillary bodies also consistently disrupt T-maze alternation (Fig. 3), although the deficits in rats often appear less severe than those after anterior thalamic lesions (Aggleton et al., 1990, 1995a; Beracochea et al., 1989; Neave et al., 1997; Vann and Aggleton, 2003). This severity difference may reflect the contribution of the direct hippocampal inputs to the ATN, which bypass the mammillary bodies (see Fig. 4). At the same time, mammillothalamic tract lesions, which disconnect the mammillary body projections to the anterior thalamic nuclei, again impair T-maze alternation (Vann and Aggleton, 2003; Vann, 2010, 2013). This disconnection finding is particularly informative as the mammillothalamic fibres go almost exclusively to the anterior thalamic nuclei. As a consequence, these results strongly reinforce the view that ATN damage is sufficient to impair T-maze alternation (see Section 2.3). Of further note is the finding that alternation deficits are also seen after lesions in the ventral part of the tegmental nucleus of Gudden (Vann, 2009), which innervates the medial mammillary body nucleus. These results imply that the mammillary body involvement in spatial alternation is not just due to its hippocampal inputs, a view directly supported by disconnection studies (Vann et al., 2011).

Not all sites densely connected with the anterior thalamic nuclei produce marked spatial alternation deficits when lesioned. The retrosplenial cortex provides an intriguing example as it is densely interconnected with both the hippocampus and the anterior thalamic nuclei (Van Groen and Wyss, 1992a, 2003; Vann et al., 2009), yet under standard testing conditions, retrosplenial cortex lesions can sometimes spare T-maze alternation (Aggleton et al., 1995b; Neave et al., 1994; Pothuizen et al., 2008) as well as radial arm-maze performance (Aggleton, 2010; Alexinsky, 2001; Vann et al., 2003). Radial-arm maze deficits are, however, unmasked when the lesions are made unusually extensive (Vann and Aggleton, 2002) and when rats are forced to rely on distal visual cues to solve the task (Vann et al., 2003). Likewise, for T-maze alternation, retrosplenial lesion effects only clearly emerge when the strategies available to the animal are constrained by the experimenter, e.g., when animals are increasingly forced to rely on direction information to solve the task (e.g., Pothuizen et al., 2008). The implication is that retrosplenial cortex lesions only disrupt some aspects of spatial learning (Vann et al., 2003; Aggleton, 2010; Hindley et al., 2014), so leaving the animal able to compensate by using other strategies. This conclusion is supported by the finding that temporary retrosplenial inactivation causes T-maze alternation deficits that seem appreciably more severe than those associated with conventional lesions (Nelson, unpublished findings).

Another efferent target of both the anterior thalamic nuclei and the hippocampus is the medial prefrontal cortex. Lesions in this cortical region give a mixed profile of effects on spatial learning. Studies often find mild or even no alternation impairment (e.g., Aggleton et al., 1995b; Granon and Poucet, 2000; Sánchez-Santed et al., 1997; Shaw and Aggleton, 1993), while performance can recover with additional training (Brito et al., 1982). There is also consistent evidence that medial prefrontal damage increases perseverative behaviour, making it more difficult to switch spatial strategies (Dias and Aggleton, 2000; Ragozzino et al., 1999). It appears, therefore, that the principal alternation deficits following medial prefrontal damage are not spatial per se, although they have the potential to impact on spatial memory tasks (Dias and Aggleton, 2000).

Finally, the cingulum bundle contains fibres linking a wide variety of cortical and subcortical sites, including the cingulate and prefrontal cortices, as well as the anterior thalamic nuclei and hippocampus (Domesick, 1970; Mufson and Pandya, 1984;Fig. 3). Cutting this bundle in rats impairs T-maze alternation (Aggleton et al., 1995b; Neave et al., 1997; Warburton et al., 1998). While this result is consistent with the pattern of limbic interactions revealed by other lesion studies, it cannot be determined which fibre connections within the cingulum bundle are most critical for alternation performance. Even so, the array of alternation deficits in sites directly linked with the anterior thalamic nuclei reinforces the view that this thalamic region comprises part of a distributed system that supports spatial learning.

## 4 Evidence for covert pathology after anterior thalamic lesions

A question arising from the lesion studies concerns the manner in which these various sites depend on each other. This issue has already been partly considered in the description of disconnection studies between the anterior thalamic nuclei and the hippocampus. A different way of addressing this issue is to examine how anterior thalamic damage affects neural functioning in other brain areas. Such studies provide growing evidence that ATN lesions cause profound dysfunctions in an array of limbic brain structures, dysfunctions that often seem undetectable by standard histological means. One example, concerning hippocampal neurogenesis, has already been mentioned in Section 3.1.

### 4.1 Mapping changes in immediate-early gene (IEG) expression following anterior thalamic lesions

Immediate-early gene (IEG) expression can provide an indirect marker of neuronal activity (Dragunow and Faull, 1989; Guzowski, 2002; Herdegen and Leah, 1998). For this reason, IEG imaging has been used to gain insights into the neural correlates of learning in the normal brain (Aggleton et al., 2012; Dragunow and Faull, 1989; Guzowski et al., 2001; Tischmeyer and Grimm, 1999). This same imaging technique can also be used to map the consequence of lesions in target brain sites. Two IEGs that have been studied in particular detail are c-*fos* and *zif268* (the latter also known as EGR1 and NGFI-A). While the expression of both of these IEGs has repeatedly been linked to spatial learning, including T-maze alternation and radial-arm maze working memory (Guzowski et al., 2001; Guzowski, 2002; He et al., 2002; Jenkins et al., 2004a; Nagahara and Handa, 1995; Vann et al., 2000a,b), c-*fos* and *zif268* have different expression dynamics and regulatory functions (Guzowski et al., 2001; Kubik et al., 2007; Tischmeyer and Grimm, 1999).

**Fig. 5:**
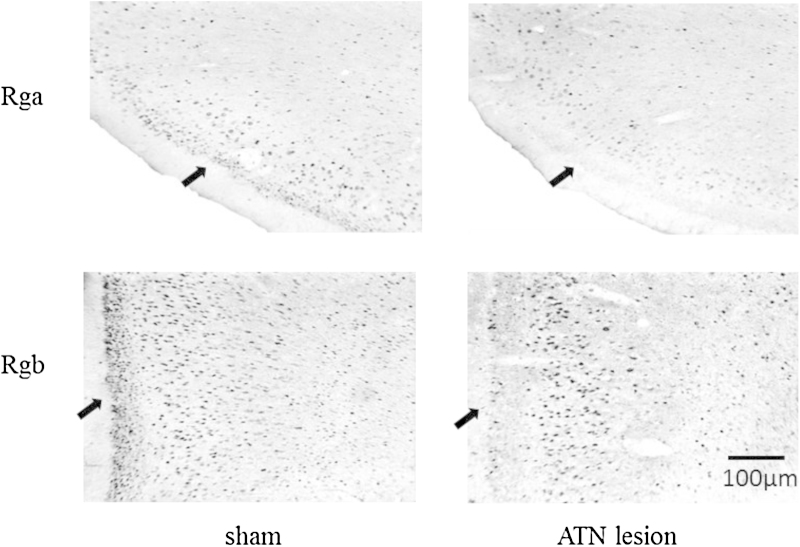
Photomicrographs of brightfield coronal sections showing the extent of Fos protein staining (dark cells) in the retrosplenial cortex in rats with anterior thalamic lesions (ATN) or sham surgeries. The sections are taken from two subareas within the granular retrosplenial cortex (Rga and Rgb). The arrows highlight the dense Fos-positive staining in layer II in the sham controls, which contrasts with the marked depletion of Fos in the same layer in the rats with anterior thalamic lesions. The absence of Fos staining contrasts with the fact that the neurons are still present (e.g., when visualised with Nissl or NeuN staining).

Lesions in the ATN decrease c-*fos* expression in a wide array of limbic and related cortical structures (Dupire et al., 2013; Jenkins et al., 2002a,b, 2004b; Poirier and Aggleton, 2009). Many of these same sites normally show increased c-*fos* activity associated with radial-arm maze performance (Vann et al., 2000a,b; see also Pothuizen et al., 2009). These affected sites include the dorsal and ventral hippocampus, retrosplenial cortex (Fig. 5), and prelimbic cortex, suggesting that anterior thalamic nuclei lesions can block the rise in c-*fos* expression associated with spatial learning (Dupire et al., 2013; Jenkins et al., 2002a; Vann et al., 2000a,b). As this lesion-induced c-*fos* hypoactivity is found in the ipsilateral hemisphere of rats with unilateral ATN lesions (Jenkins et al., 2002a,b; Poirier and Aggleton, 2009), it is not merely the consequence of any gross motor or exploratory changes produced by the surgery. The likely significance of these results is highlighted by the demonstration that inhibition of c-*fos* expression within the hippocampus (with antisense) results in a significant increase in error rates in animals performing the radial-arm maze task (He et al., 2002). The effects of ATN lesions on IEG expression are not, however, restricted to spatial tasks as some limbic sites show reductions in c-*fos* activity in home cage animals, i.e., in a baseline, resting state (Jenkins et al., 2004b).

The impact of anterior thalamic lesions on retrosplenial cortex IEG activity is particularly pervasive (Aggleton, 2008; Jenkins et al., 2002a,b, 2004b;Fig. 5). Marked decreases in c-*fos* and *zif268* expression are found irrespective of ATN lesion method or strain of rat (Dumont et al., 2012; Dupire et al., 2013; Jenkins et al., 2002a,b, 2004b; Poirier and Aggleton, 2009). Within a week of ATN surgery, very evident Fos depletions are seen in the superficial layers of the granular retrosplenial cortex (Poirier and Aggleton, 2009). When rats are examined 9–10 months after anterior thalamic surgery, the reduction in Fos positive cells is even more extensive, encompassing the dysgranular retrosplenial cortex and the deeper lamina within granular retrosplenial cortex (Jenkins et al., 2004b; Poirier and Aggleton, 2009). Detailed examination shows that these very marked changes in IEG expression are not visible with standard histological methods, e.g., Nissl stains, as there is no loss of neurons and only very subtle morphometric changes (Poirier and Aggleton, 2009). The striking IEG hypoactivity in retrosplenial cortex is of added interest as performance on the radial-arm maze task by normal rats increases both c-*fos* and *zif268* expression in retrosplenial cortex (Pothuizen et al., 2009).

The *zif268* changes following ATN lesions are not as widespread as those seen for c-*fos*. For example, ATN lesions do not appear to reduce *zif268* activity levels in the hippocampus proper or prelimbic cortex, though decreases in both c-*fos* and *zif268* activity are found in the postsubiculum (Dumont et al., 2012; Jenkins et al., 2002a,b). These findings highlight how the distal effects of ATN lesion are selective, e.g., no c-*fos* or *zif268* differences were found in somatosensory, visual or auditory cortices (Jenkins et al., 2002a; Poirier and Aggleton, 2009). The specificity of these retrosplenial IEG changes can also be seen in the finding that lesions in the laterodorsal thalamic nucleus, which projects to many of the same targets as the anterior thalamic nuclei (Van Groen and Wyss, 1992b, 2003), do not produce the same clear reductions in either c-*fos* or *zif 268* in the granular retrosplenial cortex (Poirier and Aggleton, 2009).

Despite this selectivity, the IEG changes following ATN lesions are not simply restricted to sites that receive direct inputs from these nuclei. For example, c-*fos* hypoactivity is observed in the dentate gyrus (Jenkins et al., 2002a), which is not directly innervated by the anterior thalamus nuclei. A related finding is the demonstration that mammillothalamic tract lesions cause widespread decreases in c-*fos* activity across retrosplenial cortex (Vann and Albasser, 2009). The effects of these surgeries are indirect as the fibres in the mammillothalamic tract terminate in the anterior thalamic nuclei, i.e., do not to reach the retrosplenial cortex.

Other evidence that these lesion-induced IEG reductions do not require a loss of direct inputs comes from recordings in slices of retrosplenial tissue (Garden et al., 2009). Retrosplenial brain slices taken from rats with unilateral anterior thalamic lesions showed a loss of long-term depression in the granular retrosplenial cortex in the hemisphere ipsilateral, but not contralateral, to the lesion. This absence of long-term depression was associated with a local decrease in GABA_A_ mediated inhibitory transmission (Garden et al., 2009). This selective loss of synaptic plasticity is striking not only because it occurred in those laminae showing the most marked Fos depletions after anterior thalamic lesions, but also because it provides tangible evidence that anterior thalamic lesions can induce distal pathological changes likely to affect learning processes within the retrosplenial cortex. Finally, this slice study shows that these distal changes do not merely reflect a decrease in current afferent stimulation, as in this study the stimulation is provided by the experimenter (Garden et al., 2009).

### 4.2 Other markers of distal dysfunction following anterior thalamic lesions

Van Groen et al. (1993) published the initial descriptions of retrosplenial changes following rostral thalamic lesions. In that study, the lesions reduced acetylcholinesterase, cytochrome oxidase, as well as muscarinic and 5-HT1B receptor binding in the retrosplenial cortex of rats. The thalamic lesions were, however, quite extensive (Van Groen et al., 1993) and so included other rostral thalamic nuclei, leaving the need for studies with more selective thalamic surgeries.

Microarray techniques have been used to capture the extent to which more selective anterior thalamic damage changes granular retrosplenial cortex gene expression (Poirier et al., 2008). This study revealed that, following anterior thalamic lesions, the retrosplenial cortex undergoes pervasive cellular transcriptome changes, including lower relative levels of specific mRNAs involved in energy metabolism and neuronal plasticity. These changes in functional gene expression may be largely driven by decreases in the expression of multiple transcription factors, including *brd8*, *c-fos*, *fra-2*, *klf5*, *nfix*, *nr4a1*, *smad3*, *smarcc2*, and *zfp9*, with a much smaller number (*nfat5*, *neuroD1*, *RXRγ*) showing increases (Poirier et al., 2008).

*In situ* hydridisation techniques have also uncovered the distal impact of ATN lesions. Unilateral ATN lesions altered the relative expression of c-*fos*, *zif268*, *5ht2rc*, *cox6b* and *kcanb2* between the intact and lesioned hemispheres in the granular retrosplenial cortex (Amin et al., 2010). With the exception of *cox6b*, these changes reflected a marked reduction in layer II of the granular retrosplenial cortex ipsilateral to the anterior thalamic lesion. Only *cox6b* showed a different pattern, with lower expression in the deeper layers (V) and relatively higher expression in lamina II (Amin et al., 2010).

Recent immunohistochemical studies have revealed the differential effects of anterior thalamic lesions on c-AMP response element binding protein (CREB) and phosphorylated CREB (pCREB) in the hippocampus (Dumont et al., 2012; see also Dupire et al., 2013). While hippocampal pCREB levels were significantly lower in animals with ATN lesions, hippocampal CREB levels appeared unaltered. These different profiles are intriguing because the conversion of CREB to pCREB within the hippocampus plays a key role in the consolidation of spatial learning (e.g., Guzowski and McGaugh, 1997; Mizuno et al., 2002; Silva et al., 1998). The implication is that the conversion of CREB to pCREB within the hippocampus is partly under the control of the anterior thalamic nucleus inputs (Dumont et al., 2012), although basal levels of CREB remain to be assessed. Reductions of pCREB following ATN lesions have also found in the granular retrosplenial cortex, as well as in the hippocampus (Dumont et al., 2012; Dupire et al., 2013).

Endogenous long-term brain metabolic capacity within the granular retrosplenial cortex is compromised by anterior thalamic damage. One source of evidences comes from studies of cytochrome oxidase, a marker of cell metabolism. The initial finding of reduced cytochrome oxidase activity in the retrosplenial cortex (Van Groen et al., 1993) has been replicated with more selective, cytotoxic anterior thalamic lesions (Mendez-Lopez et al., 2013). The reductions in cytochrome oxidase activity were largely restricted to the superficial laminae of the retrosplenial cortex and did not extend to the hippocampus (Mendez-Lopez et al., 2013). This evidence of reduced retrosplenial metabolic activity complements findings from microarray studies (Poirier et al., 2008).

A recent study of the functional consequences of these distal changes (Dupire et al., 2013) took advantage of the finding that environmental enrichment improves the learning performance of rats with ATN lesions on a variety of spatial tasks (Dupire et al., 2013; Loukavenko et al., 2007; Wolff et al., 2008b). Rats with ATN lesions were given an enriched environment in the home cage for 25 days. Despite clear improvements in spatial learning, when compared to rats with ATN lesions that were standardly housed, the rats with ATN lesions receiving an enriched environment still showed abnormal levels of Fos and pCREB in the retrosplenial cortex and ventral subiculum (Dupire et al., 2013). Consequently, the behavioural recovery was not paralleled by a recovery in these limbic molecular markers, a result that could be interpreted as showing that these distal molecular changes are not linked to the learning deficits. Before reaching this conclusion, it is important to remember that the enriched groups with ATN lesions remained impaired with respect to their ‘enriched’ sham surgical groups. Thus, although the improvement in learning associated with environmental enrichment happened in spite of the distal molecular changes, an underlying learning deficit remained.

Although environmental enrichment appears to mitigate some effects of ATN lesions on tests of spatial memory without any concomitant recovery in Fos levels in distal sites, this partial recovery in behavioural function may be mediated by other molecular changes that are induced by environmental enrichment. It is know that environmental enrichment promotes changes in an array of genes linked to neuronal structure, synaptic plasticity and transmission (e.g., Rampon et al., 2000). Similarly, recent evidence has shown that environmental enrichment can lead to the recruitment of additional areas into the network of structures that support learning and memory (e.g. Bonaccorsi et al., 2013; Leger et al., 2012). It is, therefore, possible that such changes underlie the partial recovery in performance on tests of spatial memory in rats with ATN lesions given an enriched environment, despite the persistence of Fos hypoactivity in the retrosplenial cortex and other distal sites.

## 5 Explaining the spatial deficits following anterior thalamic lesions

The Introduction posed the question of whether ATN lesions result in multiple deficits that combine to disrupt spatial alternation. At first sight the demands of the T-maze alternation task appear straightforward, but in fact the task is highly complex as it lends itself to multiple spatial strategies, along with various nonspatial demands that could prove vulnerable to ATN damage. It transpires that the many demands of the alternation task are paralleled by the multiple anterior thalamic nuclei, which are thought to have different functions reflecting their different connectivities (Fig. 2). A simplistic overview would be that the anterodorsal nucleus helps to provide direction information, the anteroventral nucleus helps to encode place information, while the anteromedial nucleus is important for response flexibility and temporal information (Aggleton et al., 2010). Consequently, the additive effects of damage to all three nuclei could be catastrophic, affecting alternation behaviour in a number of ways. Indeed, as would be predicted, separate selective lesions centred in the anteromedial nucleus and in the anteroventral nuclei produce only limited T-maze alternation deficits, which are considerably more severe when the lesions are combined (Aggleton et al., 1996).

One way to test this multiple function hypothesis would be to study the effects of lesions confined to individual anterior thalamic nuclei and to focus on tasks that are much more constrained in their spatial demands. The latter point addresses the fact that most standard tests of spatial learning are highly complex. A task such as learning to find a fixed location in the Morris water-maze involves learning procedural rules (Bannerman et al., 1995; Cain et al., 2006), as well as requiring effective navigation skills (Whishaw et al., 1995). Furthermore, it is often assumed that rodents in the Morris water-maze learn the absolute platform location with respect to distal room cues, yet there is evidence that rats may learn heading, rather than place, information (Hamilton et al., 2007; see also Horne et al., 2012). One solution is to employ tasks in which cue types are unusually controlled, leaving it easier to define the nature of the spatial strategy being adopted.

In one example, rats were trained to swim to the corner of a rectangular pool that was defined by the geometric relationship of the adjacent walls (Aggleton et al., 2009). The correct corner was specified by having a long wall to the left and a short wall to the right. (These criteria describe the two diametrically opposite corners in the pool). Other spatial cues were excluded by rotating the maze after every trial and by using a curtain to block distal room cues. Rats with ATN lesions were impaired at learning this problem, whether repeatedly trained to swim to the escape corner or repeatedly placed on the escape platform prior to swimming in the pool for the first time (Aggleton et al., 2009; Dumont et al., 2014b). These same rats did not appear to recognise the correct corner when it was reached on a probe trial. Hippocampal lesions also impair the ability to discriminate the relative lengths of a pool (McGregor et al., 2004).

Studies using other constrained spatial problems have shown that rats with ATN lesions are impaired at ‘path integration’, i.e., are poor at plotting and updating their route in the dark using interoceptive cues (Frohardt et al., 2006). Rats with ATN lesions also failed to locate a hidden platform set at a constant distance and direction from a moving beacon (Wilton et al., 2001b). This deficit in direction learning is unusual as it differs from the effects of hippocampal lesions, which spare this task (Pearce et al., 1998). Together, these studies reveal the importance of the anterior thalamic nuclei for spatial problems that extend beyond allocentric location learning (see also Aggleton et al., 1991).

In a recent study that attempted to isolate the ability to use the relative positions of specific visual stimuli, i.e., allocentric cues, rats were trained in a square pool in which the correct corner was specified by contrasting patterns on the adjacent pool walls (Dumont et al., 2014b). Training was ‘passive’, in that the rat was repeatedly placed on an escape platform in the ‘correct’ corner, prior to being allowed to swim to that corner for the first time (see Horne et al., 2012). This training protocol helps to eliminate unwanted strategies, such as swimming towards a specific wall and then turning right (or left) to always reach the platform. While rats with ATN lesions failed to select the correct corner on first choice, they could recognise the correct corner once it had been reached (Dumont et al., 2014b). These same rats were severely impaired on T-maze alternation (Dumont and Aggleton, 2013). The spared place recognition shown by these rats adds further weight to the argument that difficulties in place discrimination are not sufficient to explain the full severity of the T-maze alternation deficit after ATN lesions.

A different approach is to consider those spatial tasks that appear unaffected by ATN lesions. One example, already noted, is the ability to learn an egocentric discrimination, e.g., always turn to the right for food (Warburton et al., 1997; see also Mitchell and Dalrymple-Alford, 2006; Sziklas and Petrides, 1999). A second example concerns the use of local intra-maze cues to solve a biconditional problem: If in context A (e.g., dark patterned box) select item X (e.g., digging pot with paper), if in context B (e.g., plain box) select item Y (e.g., digging pot with beads) (Dumont et al., 2014a). Rats with ATN lesions could perform this problem at normal levels, in stark contrast to the spatial biconditional deficit found when the task is: If in place A (e.g., NE corner of room) select item X (e.g., digging pot with paper), if in place B (e.g., SW corner of room) select item Y (digging pot with beads) (Dumont et al., 2014a; Sziklas and Petrides, 1999). This dissociation highlights the specificity of some ATN lesion deficits as, aside from the use of distal allocentric cues in the second task, all other features were matched.

The notion that ATN lesions induce an array of different, complementary deficits is given further support by the evidence of distal molecular changes in an array of limbic sites. These molecular disruptions point to a decrease in metabolic activity, a loss of plasticity, and a reduction in hippocampal neurogenesis. Some of the most marked changes after ATN lesions are seen in the retrosplenial cortex and hippocampus, two areas involved in multiple aspects of spatial learning. These distal lesion effects need not, however, be confined to spatial processes. There is, for example, evidence that the rat retrosplenial cortex contributes to cognitive flexibility (Nelson et al., 2014), and so this function might be indirectly affected by ATN damage. At present, the evidence that these distal changes can exacerbate ATN lesion effects remains circumstantial. Indeed, there is recent data that questions the impact of some of these ‘covert’ distal effects (Dupire et al., 2013). At the same time, the degree and nature of the lesion-induced changes observed in sites like the granular retrosplenial cortex leave it difficult to believe that they are without effect. For example, a slice recording study confirmed the loss of retrosplenial plasticity following ATN lesions (Garden et al., 2009). There remains, however, the need to test this covert pathology hypothesis more directly, e.g., by injecting antisense into the retrosplenial cortex to block the actions of c-*fos* and *zif268*.

As stated in the Introduction, two accounts for the severity of the ATN lesion effects are that the surgery either disrupts multiple facets of spatial learning or it impairs a fundamental process leading to a wide array of spatial deficits, i.e., a hierarchical effect. Up to now, this review has emphasised the former view, that the ATN comprise multiple nuclei with diverse spatial functions. It is, however, necessary to consider the more parsimonious, hierarchical account, i.e., that a principal deficit in one function leads to deficits in other functions.

The most obvious hierarchical example would be that poor allocentric place learning leads not only to difficulties in discriminating the T-maze alternation choices but consequently results in increased rates of forgetting and heightened sensitivity to proactive interference for that same, poorly encoded, information. As already explained, a deficit confined to place learning or place recognition would not account for the severity of the ATN lesion effects on T-maze alternation (see Section 2.2). Such an account would also fail to incorporate the nonspatial functions of the ATN that could also contribute to alternation performance. A different hierarchical account would be to suppose that anterior thalamic lesions cause navigational deficits, which would then cause widespread performance deficits on many behavioural tests. As ATN lesions disrupt the head direction system, effectively leading to an absence of head direction information in the hippocampal formation (Calton et al., 2003; Goodridge and Taube, 1997), this account might seem plausible (see also Frohardt et al., 2006; Wilton et al., 2001b). In fact, this explanation is unlikely for several reasons. First, severe deficits following anterior thalamic damage are still present on some spatial tasks where the navigational demands appear very limited (e.g., Dumont et al., 2014a; Wilton et al., 2001b). Second, the navigational demands, aside from direction heading, are very constrained in the T-maze. Third, lesions of the lateral mammillary nuclei, which provide the head direction information to the anterior thalamic nuclei, spare T-maze alternation (Vann, 2005).

The overall conclusion is that the impact of anterior thalamic lesions can be considered on two levels. The first relates to the loss of the anterior thalamic nuclei themselves, with the disruption of their different but complementary functions. The second level relates to the disruption of neural activity and plasticity in sites closely related to the anterior thalamic nuclei, most notably the retrosplenial cortex and hippocampus. A consequence is that tasks like T-maze alternation are particularly sensitive to ATN damage because they make demands on diverse cognitive skills, reflecting not only the multiple anterior thalamic nuclei but also the varied, distal dysfunctions such lesions can induce. One implication of this model is that lesion studies of the ATN may overestimate those functions that are dependent on these thalamic nuclei. For this reason, there is particular value in understanding the electrophysiological properties of the anterior thalamic nuclei in awake rats (Albo et al., 2003; Jankowski et al., 2014; Taube, 1995; Tsanov et al., 2011a,b,c; Vertes et al., 2001) and how these properties might add to hippocampal functions (Jankowski et al., 2013; Vertes et al., 2004). A particularly relevant example of the latter would be to determine whether the anterior thalamic nuclei influence the hippocampal unit firing that predicts the alternation choice about to be made in the stem of a T-maze (Wood et al., 2000). This example highlights the more general need to characterise fully the classes of information associated with neuronal activity in the anterior thalamic nuclei of freely behaving rodents and to set that information within the context of a network of interdependent structures for spatial learning and memory.
